# Combined Exposure to Multiple Mycotoxins: An Example of Using a Tiered Approach in a Mixture Risk Assessment

**DOI:** 10.3390/toxins14050303

**Published:** 2022-04-26

**Authors:** Annick D. van den Brand, Bas G. H. Bokkers, Jan Dirk te Biesebeek, Marcel J. B. Mengelers

**Affiliations:** National Institute for Public Health and the Environment (RIVM), 3721 BA Bilthoven, The Netherlands; bas.bokkers@rivm.nl (B.G.H.B.); jan.dirk.te.biesebeek@rivm.nl (J.D.t.B.); marcel.mengelers@rivm.nl (M.J.B.M.)

**Keywords:** combined exposure, MCRA, mixtures, mycotoxins, relative potency factors

## Abstract

Humans are exposed to mycotoxins on a regular basis. Exposure to a mixture of mycotoxins may, therefore, result in a combination of adverse effects, or trigger the same effects. This should be accounted for when assessing the combined risk of multiple mycotoxins. Here, we show the outcome of using different approaches in assessing the risks related to the combined exposure to mycotoxins. We performed a tiered approach using assessment groups with a common target organ (kidney, liver and haematologic system), or a common adverse effect (phenomenon) (reduced white blood cell count), to combine the exposure to mycotoxins. The combined exposure was calculated for the individuals in this assessment, using the Monte Carlo Risk Assessment (MCRA) tool. The risk related to this combined exposure was assessed using toxicological reference values, e.g., health based guidance values. We show that estimating the combined risk by adding the single compounds’ risk distributions slightly overestimates the combined risk in the 95th percentile, as compared to combining the exposures at an individual level. We also show that relative potency factors can be used to refine the mixture risk assessment, as compared to ratios of toxicological reference values with different effect sizes and assessment factors.

## 1. Introduction

Mycotoxins are secondary metabolites produced by fungi. Secondary metabolites are compounds that are not essential to fungi, but can be important for the survival of the fungus [1]. Humans are often exposed to mycotoxins via contaminated food [2]. In general, mycotoxins can enter the food supply chain via fungi that invade agricultural crops in the field, or during storage and processing [3]. Fungi of the *Fusarium* genus are mostly associated with crop contamination in the field, whereas fungi of the *Aspergillus* and *Penicillium* genera are often associated with contamination during storage [4].

Being often part of the fungi’s defence and survival mechanism, the secondary metabolites may exert adverse effects on other fungi, plants, bacteria (penicillin), animals and humans [5]. Hundreds of different mycotoxins exist, because of a great variety of different fungal genera and fungal species [2]. Some of the more frequently occurring and better known mycotoxins in food are regulated under Commission Regulation (EC) No 1881/2006, such as, among others, deoxynivalenol (DON) in wheat, fumonisin B1 (FB1) in maize and aflatoxins in (ground) nuts and dried fruit [6]. Since a single fungal species may also produce a variety of different mycotoxins, the simultaneous exposure in our total diet is likely [7,8,9,10]. For example, co-occurrence of mycotoxins was observed in 75% of cereal-based products analysed in a Portuguese study [11]; co-occurrence of three mycotoxins was observed in 74% of wheat samples from Brazil (but this varied between sampling years) [12], and co-occurrence of mycotoxins with glucoside conjugates has been reported with a maximum of 49% in Korea [13]. Next to its derived critical effect, high exposure to a single mycotoxin (or a chemical in general) may exert multiple, other adverse effects [1]. Exposure to a mixture of structurally related and unrelated mycotoxins may, therefore, result in a combination of adverse effects or trigger the same effects due to dose addition. In the past, risk assessment of dietary exposure to mycotoxins focussed on single compounds and adverse outcomes based on the most critical effect. Nowadays, combined exposure to mycotoxins that show similar effects, which are not necessarily the critical effect, is gaining more and more attention.

### 1.1. Hazard Index

To assess the combined risk of exposure to multiple compounds, a tiered approach can be applied to minimize the time and effort spent on the assessment. A first indication of the risk of multiple compounds can be obtained via a hazard index (HI) approach. This approach allows one to assess the combined risk by summing the compounds’ hazard quotients (HQs), where the HQ of compound i is obtained by dividing its exposure (Exp*_i_*) by its health based guidance value (HBGV_i_) (Equations (1) and (2)):

(1) $${\mathrm{HQ}}_{i}=\frac{{\mathrm{Exp}}_{i}}{{\mathrm{HBGV}}_{i}}$$

(2) $$\mathrm{HI}=\sum_{i=1}^{n}{\mathrm{HQ}}_{i}$$

This approach may, however, overestimate a possible risk when the HBGVs of the individual compounds are based on different critical effects [14].

### 1.2. Target Specific Hazard Index (mRPI)

In higher tiers, refined assessment groups can be established. Compounds are grouped based on a common target organ or a common phenomenological effect, which may not be the critical effect for each compound in the mixture [8,14,15,16,17,18]. Vejdovszky et al. [15] proposed a modified reference point index (mRPI) approach to combine the risk of compounds with a common target organ, which can also be applied to compounds with a common phenomenological effect. Based on the toxicity data of the effect on the organ (e.g., the kidney or reproductive system) or phenomenon (e.g., tubular cell degeneration) a reference point (RP) can be determined for every individual compound. This RP, also known as point of departure, was compared to the respective compound’s exposure, taking different assessment factors (AFs) into account. By doing so, a reference point quotient (RPQ) is obtained that is comparable to a HQ (Equations (3) and (4)), but with the difference that the RPQs are restricted to the same target organ or phenomenon, which may not be the case for the HQs. To combine the exposure and assess the combined risk of the chemicals, the default approach of dose addition was assumed [17,19].

(3) $${\mathrm{RPQ}}_{i}=\frac{{\mathrm{Exp}}_{i}{*\mathrm{AF}}_{i}}{{\mathrm{RP}}_{i}}$$

(4) $$\mathrm{mRPI}=\sum_{i=1}^{n}{\mathrm{RPQ}}_{i}$$

Summing the RPQs of each compound (i), therefore, results in a risk metric (mRPI) that is not (solely) based on a HBGV, and is more refined than the HI.

Combining the risk of multiple compounds with an effect on a common target organ may, however, overestimate a possible risk. For example, from a toxicological point of view, an effect on glomerular filtration is not comparable to dysfunctional proximal tubular reabsorption, and the assumption of dose addition may, therefore, not always apply. Grouping the compounds in a higher tier based on a common phenomenological effect will, therefore, provide a more accurate estimation of the combined risk of that common phenomenon.

### 1.3. Relative Potency Factors

Usually, a limited number and often different types of (animal) studies are available for grouping compounds in an assessment group based on a common phenomenological effect and for deriving their respective RPs and related AFs. The ratios between those values (the ratio between the RP divided by the AF of the compounds) are sometimes used to express the exposure to compounds in an assessment group as exposure-equivalents of a reference, or index, compound [20]. By doing so, the exposure to multiple compounds can be combined. However, single RPs may not accurately reflect the equivalence between compounds, resulting in differences in study duration, species used and the effect sizes corresponding to the RPs. Adjusting RPs for these differences by applying default AFs may introduce more or other uncertainties regarding the equivalence between the compounds for a certain effect. For example, because the "true" difference between man and animal of a compound may be less or more than the applied default AF, and may differ between compounds. This methodology of expressing exposure-equivalents using ratios of AF-adjusted RPs is sometimes referred to as relative potency factors (RPFs) (e.g., [20]), but is more comparable to the mRPI approach (Equations (3) and (4)).

The RPF methodology has previously been applied to several other groups of compounds, such as dioxins and dioxin-like polychlorinated biphenyls (PCBs), organophosphorus, N-methyl carbamate pesticides, and per- and polyfluoroalkyl substances [21,22,23,24]. Expressing the exposure in equivalents of the reference compound (RefEQs) using RPFs, and comparing these equivalents to a guidance value of the reference compound, is thought to be a more precise approach to assess the risk of combined exposure (see Equation (5)).

(5) $$\mathrm{RefEQs}=\sum_{i=1}^{n}{\mathrm{Exp}}_{i}{*\mathrm{RPF}}_{i}$$

where i is a compound. The RPF of the reference compound equals 1.

In this paper, we did not consider the ratios of (AF-adjusted) RPs as RPFs, as sometimes used by the European Food Safety Authority (EFSA) [20], because this approach does not meet the conditions needed to derive unbiased relative potencies. To derive RPFs, several conditions must be met: (1) the same endpoint should be considered, (2) the dose response curves should be parallel (on log scale), (3) the compounds should not interact, i.e., dose addition applies, and (4) RPFs should be derived from experiments with (very) similar experimental setups to avoid differing setups and conditions that influence the derived potency differences [22,24,25]. The second condition is of importance because only when curves are parallel, the RPF is not dose-dependent, i.e., one and the same RPF can be applied to all doses of a compound to derive its equivalent reference compound dose.

### 1.4. Further Refinement

**Figure 1:**
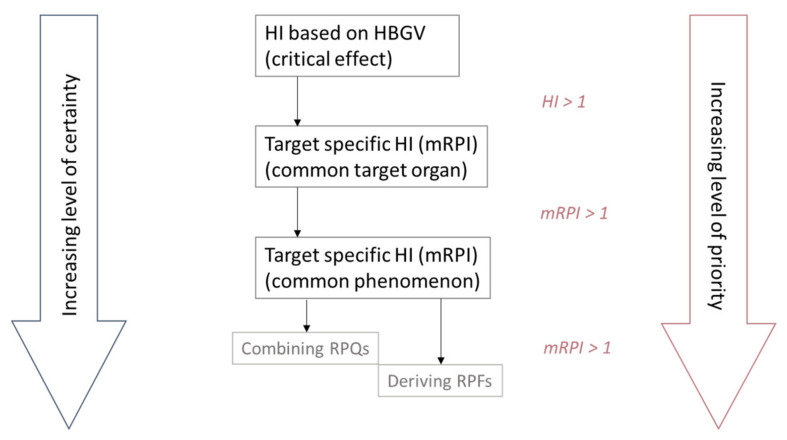
Overview of the different approaches to combine the exposure and assess the risk of multiple compounds. HI; hazard index; HQ: hazard quotient; mRPI: modified reference point index; RPQ: reference point quotient; RPF: relative potency factors.

In general, applying higher tier assessments requires more standardized data on the same effects for all compounds that need to be assessed. To minimize the time and effort spent on a mixture risk assessment, an HI approach can be used as a first estimate. However, an HI can be based on HBGVs that do not correspond to the same critical effect. An mRPI approach requires at least toxicological information on a common target organ or effect, and the proposed RPF approach requires (parallel) dose–responses for the same endpoint from experiments with similar setup. Sufficient data to proceed to a higher tier, and thereby refining the risk estimate, may not always be available for each compound in the group. As a consequence, higher tier assessments may encompass fewer compounds or may be considered not feasible due to data limitation. On the other hand, in data-rich situations, information regarding mechanism of action or adverse outcome pathways can also be used to further refine the grouping of multiple compounds, as has been done in the case of dioxins and dioxin-like PCBs. Figure 1 shows an overview of the aforementioned approaches in a tiered mixture risk assessment.

In this study, we show the outcomes of using the different approaches in assessing the risks related to the combined exposure to multiple compounds. As an example, we use the data that were used to calculate the exposure to single mycotoxins in the Netherlands, as previously estimated using (mycotoxin-dedicated) total diet studies (TDS) for 1–2 year olds by Pustjens et al. [26] and 2–6 and 7–69 year olds by Sprong et al. [27]. This study should, therefore, be considered as a methodological example of a mixture risk assessment, rather than a risk assessment of the current combined exposure to mycotoxins, as the data used in this assessment are not the most recent data available.

## 2. Results and Discussion

To assess the risks of multiple compounds, a tiered approach can be applied to minimize the time and effort spent on the assessment. Recently, a risk-based prioritisation of chemicals was proposed for grouping. When a compound does not contribute more than X% to its individual HBGV, it is likely that its relative contribution to a combined risk of the mixture is low and thus of low priority for grouping [14,28]. In the case of the mycotoxins, an HBGV was not available for every compound and the HI approach and risk-based prioritisation of mycotoxins for assignment into assessment groups could not be applied. Therefore, we directly proceeded to the mRPI approach for common target organs, followed by an mRPI approach for the common phenomenon.

For the mRPI approach, we created different assessment groups to assess the risks of combined exposure to the mycotoxins that were analysed in the previously published Dutch TDSs. For every mycotoxin in every assessment group, a reference point (RP) was identified, based on the study describing the lowest dose for an effect related to the assessment group (Section 2.1). This RP was subsequently combined with assessment factors (AFs), comparable to the mRPI approach from Vejdovszky et al. [15,16], to obtain a toxicological reference value (TRV). These values can be compared to the estimated exposure levels to obtain a reference point quotient (RPQ) for single mycotoxins, or a risk quotient for the combined exposure to these compounds (mRPI) (Section 2.1.4). Subsequently, different approaches to refine the assessment at the common phenomenon tier were explored (Section 2.2).

### 2.1. Assessment Group at Common Target Organ Level

First, mycotoxins with an effect on a common target organ were grouped. This was done based on literature identified in EFSA Scientific Opinions on the mycotoxins included in the Dutch TDSs, risk assessment reports from other regulatory institutions, or literature derived after the publication dates of these opinions or other risk assessment reports. From these studies, a reference point (RP) was derived and combined with assessment factors to establish a TRV for the mycotoxins in the common target organ assessment group. A TRV is not necessarily related to the critical effect, but it can be (and can, therefore, be the same as the respective HBGV). Grouping at the common target organ level is accompanied by high uncertainty, as not every reference point is based on the same effect in a common target organ. Therefore, grouping using common target organs can be considered as a prioritisation step for assessment groups. When the combined risk to a target organ gives no reason for concern, there is no need to refine the risk assessment. In this study, we used kidney, liver and the haematological system as examples of common target organs.

#### 2.1.1. Kidney

Citrinin (CIT), fumonisin B1-3 (FB1-3), nivalenol (NIV), ochratoxin A (OTA) and patulin (PAT) were grouped together as mycotoxins that are relevant for adverse effects on the kidney based on the identified studies. No evidence is available on possible kidney effects caused by the other mycotoxins considered in the TDSs. Table 1 shows the characteristics of the studies and derived TRVs of the included mycotoxins.

Citrinin: The kidney is the target organ for citrinin [29]. A 90-day rat study by Lee et al. [30] was used to derive a reference point for CIT by EFSA [29]. Although no nephrotoxic effect was observed in rats, the highest dose tested in that study (no observed adverse effect level (NOAEL) = 0.02 mg/kg bw/d) was used as a reference point by EFSA in combination with AFs for extrapolation of study duration (AF = 2) and inter- and intraspecies differences (AF = 10x10) to derive a level of no concern for nephrotoxicity: 0.1 µg/kg bw/d [29]. That level was used as a TRV in our study to assess the risk of adverse effects on the kidney after exposure to CIT. Hayashi et al. [31] did not observe kidney toxicity in mice exposed at a level of 4.5 mg CIT/kg bw/d (30 ppm) for 90 days, the highest dose tested, but this study was published after the EFSA Opinion and, therefore, not included in the EFSA Scientific Opinion previously discussed. The NOAEL from Hayashi et al. [31] appears rather high, especially since Singh et al. [32] reported signs of nephrotoxicity after oral administration of multiple doses from 1 ppm (mg/kg) up to 5 ppm of CIT for 70 days in rats. Histopathological sections of the kidney showed apoptotic cells, in particular in the proximal convoluted tubules, which were not present in the control and lowest dose group animals (of 1 ppm) [32]. However, quantitative data on these adverse effects on the proximal tubules were not reported and, therefore, it was decided to use the level of no concern for nephrotoxicity that was derived by EFSA as a TRV.

FB1-3: Kidney lesions were also observed in chronic and sub-chronic studies with rats after oral exposure to FB1 [33,34]. The Scientific Committee for Food previously derived a tolerable daily intake (TDI) of 2 µg/kg bw/d for FB1 based on the NOAELs derived from these studies, after the application of a default AF of 100 [35]. This TDI was also used in this study as the TRV to assess the risk of FB1 on adverse effects on the kidney. Considering the structural similarity of FB1-3 and the group HBGV for FB1-3 (for effects on the liver), this TRV was also applied to the other forms of fumonisins in the assessment.

NIV: Among other effects, reduced kidney weight was observed in mice fed up to 3.5 mg NIV/kg bw/d [36,37]. The dose at which no significant effect on kidney weight was described, 0.7 mg/kg bw/d, was used in this study as an RP. Since this effect was observed in a chronic, 2-year study, the AF that was applied was 100 for inter- and intraspecies differences (AF = 10 × 10).

OTA: Recently, a lower limit of the benchmark dose related to 10% extra risk (BMDL_10_) of 4.73 µg/kg bw/d for non-neoplastic kidney lesions was derived by EFSA, after exposure to OTA in pigs [38,39]. This BMDL_10_ was used as an RP in this study, and in combination with the AFs for extrapolation of study duration (AF = 2) and inter- and intraspecies differences (AF = 10 × 10), a TRV of 0.024 µg/kg bw/d was derived.

PAT: Impairment of kidney function was observed after exposure to PAT in rats in a sub-chronic study [40]. The NOAEL from that study was used as an RP, in combination with the assessment factors for extrapolation of study duration (AF = 2) and inter- and intraspecies differences (AF = 10 × 10), to derive a TRV of 4 µg/kg bw/d to assess the risk of adverse kidney effects after exposure to PAT.

**Table 1:** Assessment group nephrotoxicity.

Mycotoxin	Effect	Critical Effect	Reference Point (RP) µg/kg bw/d	RP Type	Species	Study Duration	AF	TRV µg/kg bw/d	Ref.
Citrinin	No toxicologically significant alterations in liver and kidney were observed; the authors concluded that 20 μg citrinin/kg bw/d was not nephrotoxic	Yes	20	NOAEL	Rat	90 days repeated dose	10 × 10 × 2	**0.1**	[29,30]
Fumonisin B1-3	Kidney lesions (evidence of apoptosis and increased cell proliferation of the renal tubule epithelium/hyperplasia renal tubule epithelium)	No (megalocytic hepatocytes by EFSA)	250/200	NOAEL	Rat	Chronic/sub-chronic	10 × 10	**2**	[35]
Nivalenol	Decreased kidney weight	No (reduced WBC by EFSA)	660	NOAEL	Mouse	Chronic	10 × 10	6.6 *	[36]
Ochratoxin A	Increased incidence of non-neoplastic kidney lesions	Yes	4.73	BMDL_10_	Pig	90 days repeated dose	10 × 10 × 2	**0.024**	[38,39]
Patulin	Slight impairment in kidney function	No (impaired growth by JECFA)	800	NOAEL	Rat	90 days repeated dose	10 × 10 × 2	4 *	[40]

AF: assessment factor, TRV: toxicological reference value, NOAEL: no observed adverse effect level, BMDL_10_: lower limit of the benchmark dose related to 10% extra risk, WBC: white blood cell count, JECFA: Joint FAO/WHO Expert Committee on Food Additives. * TRVs not based on a previously derived HBGV and assessment factors were derived following Vejdovszky et al., 2019 [15].

#### 2.1.2. Liver

FB1-3 and zearalenone (ZEN) were grouped together as mycotoxins that are relevant for adverse effects on the liver. Other mycotoxins in the TDSs showed no evidence of liver toxicity. Table 2 shows the characteristics of the studies and derived TRVs of these mycotoxins.

FB1-3: For FB1, a BMDL_10_ of 100 µg/kg bw/d was identified by EFSA for increased incidence of megalocytic hepatocytes in a chronic feeding study in mice [41,42]. EFSA derived a TDI of 1 µg/kg bw/d after the application of an assessment factor of 100, which applied to the sum of FB1-3 and was used as a TRV to assess the risk of adverse liver effects after exposure to fumonisins.

ZEN: For ZEN, a lowest observed effect level (LOEL) of 1 mg/kg bw/d was reported regarding hepatocellular cytoplasmatic vacuolization after chronic exposure to zearalenone in rats [43]. This RP was used, in combination with the AFs for extrapolation from a lowest observed effect level to a NOAEL (AF = 3) and inter- and intraspecies differences (AF = 10 × 10), to derive a TRV of 3.33 µg/kg bw/d. That TRV was used to assess the risk of adverse liver effects after exposure to ZEN. Battilani et al. [8] also reported liver toxic effects for zearalenone based on a EuroMix case study. A NOAEL of 1000 µg/kg bw was reported for hepatocellular cytoplasmatic vacuolization in rats [8]. However, as the original reference to the case study could not be identified, a LOEL of 1000 µg/kg bw/d for hepatocellular cytoplasmatic vacuolization was selected as an RP [43].

**Table 2:** Assessment group liver toxicity.

Mycotoxin	Effect	Critical Effect	Reference Point (RP) µg/kg bw/d	RP Type	Species	Study Duration	AF	TRV µg/kg bw/d	Ref.
Fumonisin B1-3	Increased incidence of megalocytic hepatocytes	Yes	100	BMDL_10_	Mouse	Chronic	100	**1**	[42]
Zearalenone	Hepatocellular cytoplasmatic vacuolization	No (estrogenic effect EFSA)	1000	LOEL	Rat	Chronic	10 × 10 × 3	3.33 *	[43]

AF: assessment factor, TRV: toxicological reference value, BMDL_10_: lower limit of the benchmark dose corresponding to 10% extra risk, LOEL: lowest observed effect level. * TRVs not based on previously derived HBGV and assessment factors were derived following Vejdovszky et al., 2019 [15].

#### 2.1.3. Haematological Effects

Diacetoxyscirpenol (DAS), NIV, moniliformin (MON), T2 and HT2 toxin were grouped together as mycotoxins that are relevant for adverse effects on the haematological system. Other mycotoxins in the TDSs showed no evidence of haematological effects. Table 3 shows the characteristics of the studies and derived TRVs of the included mycotoxins.

DAS: DAS, as part of the pharmaceutical compound anguidine, was reported to induce leukopenia (reduced white blood cell count) in a human clinical trial [44,45]. A NOAEL of 65 µg/kg bw/d was identified by EFSA and, after the application of assessment factors for the limited duration and the intermittent dosing regimen of the human clinical trial (AF = 10) and interindividual toxicokinetic and toxicodynamic variability (AF = 10), a TDI of 0.65 µg/kg bw/d was derived by EFSA [45]. This TDI was used as a TRV in our study.

NIV: Reduced white blood cell count was also reported in a sub-chronic study in rats after exposure to NIV [46]. The BMDL_05_ that was calculated by EFSA was used as a reference point [47]. In combination with the assessment factors for extrapolation of study duration (AF = 2) and inter- and intraspecies differences (AF = 10 × 10), a TRV of 1.75 µg/kg bw/d was derived. This was used to assess the risk of haematological effects after exposure to NIV.

MON: Exposure to MON also induced haematological effects in pigs, described as decreased haematocrit and haemoglobin levels [48]. A BMDL_05_ was derived by EFSA and used as a reference point to derive a TDI of 1 µg/kg bw/d [49]. This TDI was used as a TRV in this study.

T2/HT2 toxin: In addition, T2 toxin reduced the leukocyte (white blood cell) count after subchronic exposure in rats [50]. EFSA derived a BMDL_10_ as a reference point to derive a TDI for T2 and HT2 toxin [51]. This TDI was also used in our study as a TRV to estimate the risk of haematological effects after exposure to T2 and HT2 toxin. 

Although white and red blood cells have distinctly different functions in the body, and toxicological effects on these different types of blood cells will result in distinct physiological effects (oxygen transport versus immune function), we grouped them together. In the more refined approach, effects on white blood cells can be specified and separated from those on red blood cells (see Section 2.2). This is in accordance with Boberg et al. [52], who grouped compounds at the common target organ “haematological system”, and created subgroups for the phenomenological/specific effects on “anaemia”, “thrombocytosis” and “thrombocytopenia”.

**Table 3:** Assessment group haematological effects.

Mycotoxin	Effect	Critical Effect	Reference Point (RP) µg/kg bw/d	RP Type	Species	Study Duration	AF *	TRV µg/kg bw/d	Ref.
Diacetoxyscirpenol	Expected to induce leukopenia, agranulocytosis, and anaemia	Yes	65	NOAEL	Human	Clinical study—not chronic	10 × 10	**0.65**	[44]
Nivalenol	Haematological disturbances/reduced white blood cell count	Yes	350	BMDL_05_	Rat	90 days repeated dose	10 × 10 × 2 ^1^	**1.75**	[46,47]
Moniliformin	Haematological adverse effects (as decreased haematocrit and haemoglobin levels)	Yes	200	BMDL_05_	Pig	28 day sub-chronic	10 × 10 × 2	**1**	[48,49]
T2/HT2	Haematotoxicity—reduction in leukocyte count	Yes	3.33	BMDL_10_	Rat	90 days repeated dose	10 × 10 × 2	**0.02**	[50,51]

^1^ An additional assessment factor (AF) of 1.5 was used by EFSA to derive the HBGV, in tandem with the default 10 × 10 for extrapolation from animal studies and 2 for the extrapolation of subchronic to chronic study duration. The factor of 1.5 was used due to the limitations of the available data on reproductive and developmental toxicity of nivalenol. This latter AF was omitted for the derivation of a toxicological reference value for haematological effects. AF: assessment factor, TRV: toxicological reference value, NOAEL: no observed adverse effect level, BMDL_05_ and BMDL_10_: lower limit of the benchmark dose corresponding to 5 and 10% decrease in WBC. * Assessment factors used as derived by EFSA.

#### 2.1.4. Modified Reference Point Intake (mRPI)

The exposure levels of the single mycotoxins can be found in Sprong et al. [27] and Pustjens et al. [26], with the exception of DAS and MON. The latter two mycotoxins were analysed in the TDS samples from Pustjens et al. [26], but not reported in the that publication. As we had access to both datasets and calculations from the two TDSs that were previously published by our group, we were able to assess the combined risk of the mycotoxins in the respective assessment groups by combining all individual RPQs. Vejdovszky et al. [15,16] summed the P50 (median of the risk metric distribution) and P95 (the 95th percentile of the risk metric distribution, i.e., high risk) RPQs to obtain an mRPI that characterises the combined risk associated with the P50 and P95 exposure of the population. However, it is sometimes unlikely that all highest exposed individuals will be the highest exposed to all compounds. For example, when one compound often occurs in pasta products and another in potato products, co-exposure may be less likely. Summing population exposures or RPQs of compounds in an assessment group, especially those of the highest exposure percentiles (P95), can, therefore, result in an overestimation of the risk in a combined exposure assessment. This chance of overestimation is avoided in the combined exposure assessment using the Monte Carlo Risk Assessment (MCRA) tool (https://mcra.rivm.nl/, accessed on 1 October 2021). Using MCRA, we expressed the single compound exposure as equivalent of a reference compound, after which the single exposures could be summed at an individual level (see Method Section 4.3.3 and Equation (6)).

Indeed, Table 4 shows that using the summing of the RPQs in the mRPI approach, a slightly higher risk is obtained as compared to the risk characterisation metric at the P95 of the distribution of cumulative individual exposures obtained with MCRA. This difference is not always large, especially when the risk of the combined exposure is driven by one mycotoxin. In addition, the use of TDS samples may result in a smaller deviation of the risk metric between the mRPI and the individually calculated risk, considering the inclusion of composite samples (that may consist of multiple ingredients/food products) in the analysis.

Using both approaches, it appears that the risk metric of nephrotoxicity from the combined exposure to the mycotoxins is below one in the P50 and in the high exposure percentile (P95) in the lower bound (LB) scenario in all populations. However, in the upper bound (UB) scenario of the P95, this is higher than one for all populations. This is a result of the high contribution of OTA and CIT to the combined risk. An additional analysis using the medium bound (MB) scenario, where the concentrations below the limit of detection/quantification (<LOD/LOQ) were substituted with ½ LOD/Q, resulted in a mRPI (calculated using MCRA) of 0.507 (P50) and 1.35 (P95), 0.136 (P50) and 0.261 (P95), and 0.305 (P50) and 0.842 (P95) for 1–2 year olds, 2–6 year olds and 7–79 year olds, respectively (data not shown). Considering the very large difference between the LB and UB scenario for CIT, we can conclude that this is a result of left-censored data in combination with a relatively high LOD/Q. More refinement considering the exposure assessment, for example, by using lower LOD/Qs and increasing the number of samples per food category, would be needed to better assess the risk for the nephrotoxicity assessment group. In addition, the observed individual mean (OIM) model was used to calculate the exposure in MCRA. This model may result in estimates of the fraction of the population with a usual exposure above some standard that are too high [53]. However, other models, such as the logistic normal-normal (LNN) model, were not applicable, as the (log or power) transformed data did not meet the prerequisite of being normally distributed.

The risk of liver toxicity from combined exposure to fumonisins and ZEN is below one in all cases. Therefore, there is no need to refine the assessment for this common target organ.

In contrast, the risk of haematological effects from combined exposure to mycotoxins is clearly higher than one. This holds especially true for children (1–2 year olds and 2–6 year olds), but also the 95th percentiles in the upper bound (UB) scenario for all populations. This indicates a further need for refinement of the assessment, especially considering that the estimated exposure to these mycotoxins is highly relevant, as at least NIV and the combination of T2 and HT2 toxin have been reported to co-occur, especially in oat (products) [54].

#### 2.1.5. Other Mycotoxins

Relevant in vivo studies on health effects of other mycotoxins included in the Dutch TDSs were not identified in the related EFSA Scientific Opinions or literature after those publication dates (see Appendix A). For many mycotoxins, chronic in vivo studies are scarce, and although some mycotoxins were not included in the assessment groups in this study, it does not necessarily mean that they should not be included [55,56,57]. The risks attributed to the assessment groups may, therefore, be underestimated as a result of these lacking data. New toxicity studies could reveal new information and RPs for mycotoxins regarding the specific assessment groups. As there is a societal need to reduce animal testing, new approach methodologies (NAMs) may also aid in filling these knowledge gaps or, at least, indicate the need to include compounds in specific assessment groups using novel hazard screening approaches [58].

### 2.2. Assessment Group at Common Phenomenological Effect Level

#### 2.2.1. Assessment Group at Common Phenomenological Effect Level—mRPI

Grouping the mycotoxins based on a common phenomenological effect instead of the common target organ level can be regarded as a refinement of the estimate and thus as a higher tier assessment in case the lower tier assessment (mRPI at the common target organ level) is larger than one. For the risks that were assessed for the common target organs as described above, the estimated risk at the common target organ level indicates a need for additional refinements for the nephrotoxicity and haematological effect assessment groups. For the nephrotoxicity assessment group, no common phenomenological effect could be identified, and thus refined assessment at this level could not be performed. 

Within the haematological assessment group, multiple mycotoxins were reported that specifically reduced the number of white blood cells. For these mycotoxins, i.e., DAS, NIV and T2 and HT2 toxin, the risk of combined exposure for this common phenomenological effect can be assessed. It is worth noting that Nielsen et al. [59] did not consider reduced white blood cell counts in their identification of a common assessment group at a common phenomenological effect level. The reason for this was that changes in white blood cell count are often not consistent or treatment-related, and a reduction in white blood cell counts is sometimes not a result of damage to the white blood cells, but a secondary effect to effects on other target organs [59]. However, considering that for both NIV and T2 and HT2 toxin, reduced white blood cell counts were identified as a critical effect by EFSA, we grouped them together [51,60]. 

In this higher tier assessment group, we calculated the combined exposure and risk of DAS, NIV and T2 and HT2 toxin in the same way as the common target organ assessment groups but used the TRVs for the common phenomenological effect. The estimated risk of the combined P95 exposure to DAS, NIV and T2 and HT2 toxin in the UB scenario was 16.5, 20.3 and 5.0 when the individual risk quotients were summed, and 15.6, 18.7 and 4.5 when combining the exposure probabilistically (see Appendix B—Table A1). Since the exposure to T2 and HT2 toxin is driving the risk and MON did not contribute greatly to the combined risk of the common target organ assessment group, no major difference in the combined risk of this common phenomenological effect was observed after the exclusion of MON from the assessment group.

The individual RPQ for T2 and HT2 toxin in Austrian children (6–9 years old) was calculated by Vejdovszky et al. [16] between 0.15–0.76 in the P50 and between 0.26–1.3 in the P95. The RPQs for these mycotoxins for the Dutch children in our assessment (although 2–6 years old) were much higher as a result of the assessed risk being related to another assessment group, with another common phenomenological effect. While we looked at reduced white blood cell count, which is also considered the critical effect of T2 and HT2 toxin, Vejdovszky et al. [16] considered growth retardation as the common phenomenological effect. The median dietary exposure estimation in children was, however, similar between our assessments, except for the 95th percentile, which was higher in our dietary exposure assessment: 0.21–0.38 µg/kg bw/d in the Netherlands compared to 0.03–0.13 µg/kg bw/d in Austria [15]. This indicates that the risk related to the effect of T2 and HT2 toxin on reduced white blood cell count is more urgent and needs to be considered when assessing the risk of other compounds that have an effect on reduced white blood cell count.

#### 2.2.2. Grouping at a Common Phenomenological Effect Level—RPFs

There are also uncertainties that accompany the risk assessment at the common phenomenological effect level with the mRPI approach, as not every RP (reference point) derived for this manuscript was based on a study considering the same hazard characterisation metrics and effect sizes. The TRVs (toxicological reference values) of the mycotoxins do not, therefore, truly reflect equipotent doses, and the scaling factors as used in the MCRA tool (derived from ratios between the TRVs) do not, therefore, truly reflect the potency difference between compounds. It is, therefore, uncertain to say whether a risk is underestimated or overestimated and by what magnitude.

Thus, to scientifically robustly combine the exposure to mycotoxins for this, or any, common phenomenological effect, RPFs (relative potency factors) could be used to scale the exposure of the compounds to each other [24]. To minimize the influence of differences due to experimental setup, the RPFs should preferably be derived from data of comparable studies. Table 3 shows that a reduced white blood cell count was observed in a 90-day study (in rats) for both T2 toxin and NIV. Therefore, the data from these studies could be used to perform a simultaneous benchmark dose (BMD) analysis to derive an RPF for reduced white blood cell count as described in Appendix D. For DAS, no 90-day rat study was identified that reported on the white blood cell count, and, therefore, no RPF could be derived for DAS. Ideally, this mycotoxin also should be included in the derivation of RPFs for this common effect so that it could have been included in the refined assessment. Using the datasets on reduced white blood cell counts for T2 toxin and NIV, a simultaneous BMD analysis was performed, and an RPF of 140 for T2 toxin as compared to NIV was derived, which was independent of the dose and effect size. This derived RPF for T2 toxin compared to NIV was subsequently used to estimate the combined exposure distributions using MCRA, rather than using the ratios of the respective TRVs. The RPF of HT2 toxin was assumed to be the same as that of T2 toxin, considering their grouped HBGV (health-based guidance value) [51].

It is important to note that this combined exposure and the related risk can be calculated in MCRA either by expressing the sum of T2 and HT2 toxin as NIV equivalent, by using the RPF and then adding the exposure to these NIV equivalents to the NIV exposure and comparing it to NIV’s TRV, or vice versa by expressing NIV as T2/HT2 toxin equivalent. Using one or the other mycotoxin as a reference compound, however, resulted in a difference in the calculated combined risk, as is shown in **.** Table 5. This is in contrast to the approach taken by EFSA, which states that the choice of a reference compound has no effect on the outcome of the risk [20]. However, EFSA describes the RPFs as the ratio between RPs (as we used in the mRPI approach). Indeed, in such a case, the selection of a reference compound is independent of the choice of reference compound, as the RP of the reference compound is changed in both the numerator as well as the denominator of the equation to calculate the risk [20]. Here, the RPs are not used to calculate the RPFs, and, therefore, it matters which reference compound is selected.

The discrepancy between the two risk outcomes is caused by a difference between the TRVs for NIV and T2 and HT2 toxin that is not similar to the RPF. If the ratio between those TRVs had been the same as the RPF, then the approach would have resulted in the same risk regardless of the chosen reference compound. This was not the case because of two features of the TRVs that caused the ratio of TRVs not to reflect the true potency difference between the two compounds. For one, TRVs are derived from benchmark doses corresponding to different effect sizes, i.e., the TRVs do not reflect equipotent doses. Secondly, the residual variation in the response of both underlying studies differs, which influences the width of the BMD confidence interval and thus the BMDL. That means a larger variation will result in a wider BMD confidence interval, and consequently in a lower BMDL. Therefore, even though two compounds may be equally potent, if the residual variation in experiments with these two compounds differs, this will result in different BMDLs and subsequent TRVs, since the RPFs reflect the ratio between BMDs and not the BMDLs. To reduce the influence of the selected reference compound on the outcome of the risk, one can reduce the influence of both mentioned features. This can be achieved by performing a simultaneous BMD analysis that resolves the issue of different effect sizes (see Appendix E). The compound with the least uncertain RP, i.e., with the smallest BMDL/BMDU ratio, can then be selected as a reference compound for the combined exposure assessment.

In a simultaneous BMD analysis, a benchmark response (BMR) of 10% decrease in white blood cell count was applied for both datasets, which was a toxicologically relevant response according to EFSA based on the natural variation in white blood cell counts [51]. It should be noted that a white blood cell count decrease of up to 20% may be considered non-adverse following World Health Organization (WHO) guidance for pesticide residues in food [61]. For the purpose of our study, we used the interpretation of EFSA and derived two BMDL_10_s for NIV and T2/HT2 toxin following the simultaneous BMD analysis (Table 6).

These newly derived BMDL_10_s (RPs) were used to derive associated TRVs (the RP divided by the earlier established AFs; Table 3) of NIV and T2 and HT2 toxin. When these TRVs were used to assess the risk of the combined exposure to the compounds, similarly as done in Table 5, it was observed that the risk metrics did not differ as much as previously calculated (Table 7). Thus, the selection of the reference compound in this case did not affect the outcome of the combined risk as much. In our analysis, the ratio BMDU_10_/BMDL_10_ for NIV was the smallest (Table 6) and could, therefore, be considered as the appropriate mycotoxin to select as a reference compound. Note that the new TRV for NIV is almost 2-fold the previous TRV, as it was based on a BMDL_10_ rather than a BMDL_05_.

**Figure 2:**
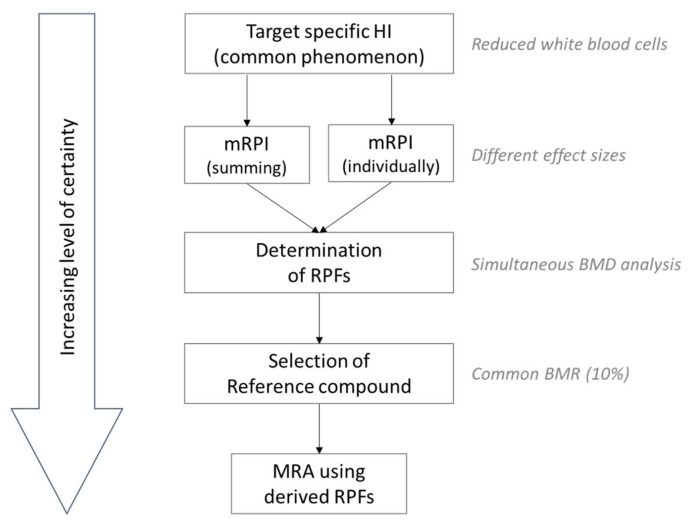
Refining the combined risk assessment of mycotoxins in the common phenomenological effect tier. HI: hazard index; mRPI: modified reference point index; RPFs: relative potency factor BMD: benchmark dose; BMR: benchmark response; MRA: mixture risk assessment.

To summarize, we showed various steps in a tiered approach to assess the risk of a combined exposure to mycotoxins based on two previously published (mycotoxin-dedicated) TDSs that were used to estimate the exposure to single mycotoxins, as summarized in Figure 2. When an indication for a risk was indicated in a lower level tier (common target organ), we continued to a higher level tier where possible (common phenomenological effect) in order to perform a more robust combined risk assessment. Ideally, the combined risk assessment is performed with TRVs that are derived based on RPs with the same hazard characterisation metrics and effect sizes. If available, RPFs can be derived and used instead (in combination with a selected, most precise reference compound) to combine the exposure to multiple compounds and subsequently calculate the respective risk. It must be noted that a high level of toxicological information is required for a combined risk assessment of multiple compounds. This information, especially when proceeding to higher level tiers, is often lacking [62]. Nonetheless, in a tiered approach, an initial estimate of risk can be obtained with little effort spent as compared to a refined approach in a higher level tier.

The current study should be considered as a methodological example of a mixture risk assessment, rather than a risk assessment of the current combined exposure to mycotoxins, as the data used in this assessment were not the most recent data available and considering the limited analytical samples obtained. Nonetheless, it is of importance to consider the combined risk when addressing the risk of mycotoxins, as the co-exposure of multiple mycotoxins is well-established all over the world [11,12,13,63,64]. Moreover, multiple mycotoxins are frequently detected in blood and urine samples from various human populations [63,65,66,67].

## 3. Conclusions

When performing a risk assessment of multiple chemicals combined, a probabilistic approach where the co-exposure of individual persons is accounted for can be considered to reduce an overestimation of the risk, compared to summing the risk metrics of the single compounds. To further refine a mixture risk assessment, the application of RPFs obtained from a simultaneous BMD analysis is a more precise way of combining the exposure than using ratios of TRVs of the respective compounds.

## 4. Materials and Methods

### 4.1. Overview of Applied Approach

In this study, mycotoxins considered in the TDSs by Sprong et al. [27] and Pustjens et al. [26] were reviewed for their common effects at the organ level or phenomenon level and wherever relevant, grouped together in assessment groups to combine their exposure and estimate the related risk. This was done following an mRPI approach, using RPs and TRVs relevant to the assessment groups. To further refine the assessment, RPFs for two mycotoxins with a common phenomenon were derived. These were used to combine the exposure to the two mycotoxins. To be able to select an appropriate reference compound, a simultaneous BMD analysis was performed to align the effect sizes that were used to derive the TRVs for these mycotoxins and estimate the related risk.

### 4.2. Hazard Identification and Characterisation

The mycotoxins included in the mycotoxin-dedicated TDSs by Sprong et al. [27] and Pustjens et al. [26] were considered for the combined risk assessment described in the present paper. These mycotoxins are listed in Table 8.

The adverse effects of the mycotoxins under consideration were identified in the literature from the respective EFSA Scientific Opinions, reports from other regulatory institutes, or literature derived after those publication dates and the OpenFoodTox database version 3 by EFSA [68]. Embase was used as a literature database, and search strings were developed in collaboration with an information specialist at RIVM. This information was used to group the mycotoxins in assessment groups with a common target organ and a common phenomenological effect.

For every mycotoxin in an assessment group, a hazard characterisation value, here identified as toxicological reference value (TRV), was derived or obtained. These TRVs were based on HBGVs identified by EFSA (if the assessment group corresponded to the respective mycotoxin’s critical effect) or based on a reference point (RP) divided by the respective derived assessment factors (if no HBGV was available for the mycotoxin that related to the specific target organ/phenomenon). The RP for a specific mycotoxin in an assessment group was obtained following identified RPs, related to that assessment group, by EFSA or other regulatory institutions. Where necessary, the same assessment factors as used by EFSA were used to adjust the RPs. The assessment factors related to the RPs not identified by EFSA were derived following the scheme presented by Vejdovszky et al. [15] that is based on EFSA’s standard conventions [69]. Although aflatoxins and sterigmatocystin are known to cause hepatocellular carcinomas and hepatic hemangiosarcomas, these mycotoxins were not included in the assessment group for liver toxicity. This manuscript only addressed compounds that are non-genotoxic or non-carcinogenic.

#### 4.2.1. Relative Potency Factor

**Figure A1:**
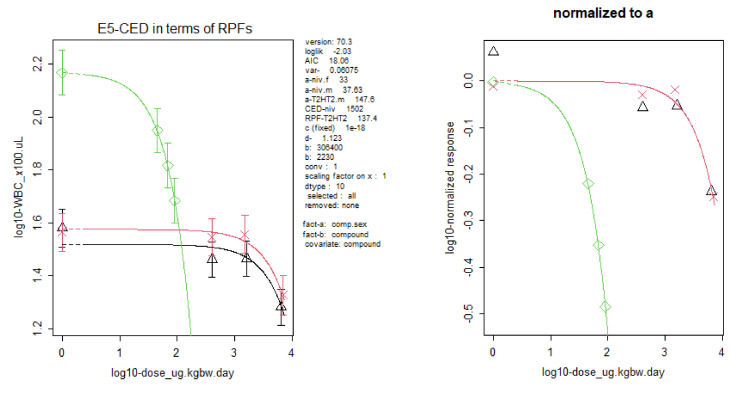
Left pane: results of the benchmark dose analysis and the derivation of the relative potency factor of T2/HT2. The RPF of T2/HT2 toxin as compared to nivalenol is 137.4 (see legend), rounded as 140. Right pane: visualization of parallel curves by plotting the results after normalization to the background. Green line: T2 toxin, red line: nivalenol (males), black line: nivalenol (females). Red and black line exactly overlap in the right pane after correcting for background levels.

The datasets of NIV and T2/HT2 toxin from [46,50] were used to derive the RPF (for T2/HT2 toxin compared to NIV. The analysis was performed using the PROAST software package version 70.3 (https://www.rivm.nl/proast, RIVM, 1 October 2021). For NIV, the standard deviations of the originally reported data were transformed to standard errors of the mean to match the variation metric of the T2 toxin dataset (Appendix D—Table A4). The RPF of HT2 was assumed to be the same to that of T2, considering their group HBGV [51]. See Appendix D (Figure A1) for a detailed description of the derivation of the RPF in this study. The derived RPF is independent of the effect size of the BMD analysis.

#### 4.2.2. Benchmark Dose Analysis

**Figure A2:**
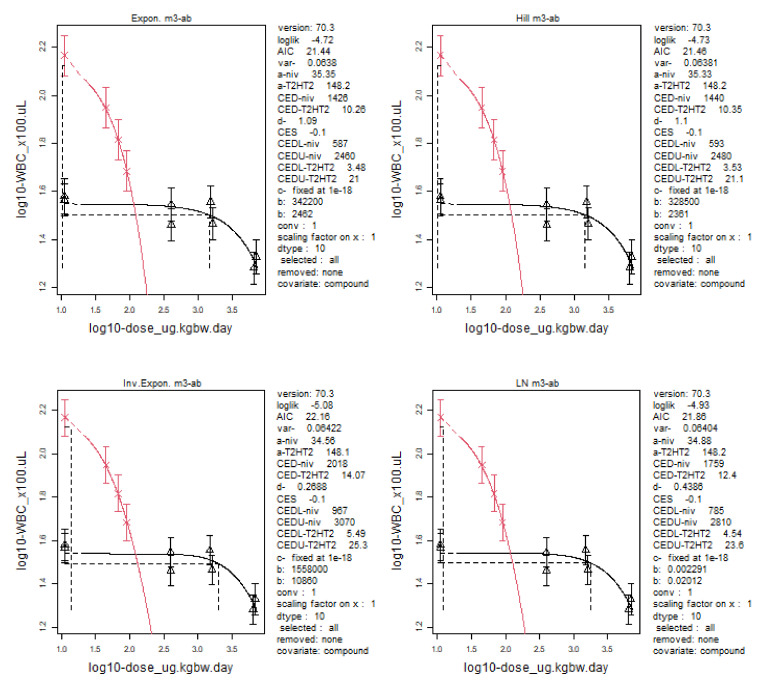
Combined benchmark dose analysis of nivalenol (black line) and T2 toxin (red line) with a benchmark response of 10%.

**Figure A3:**
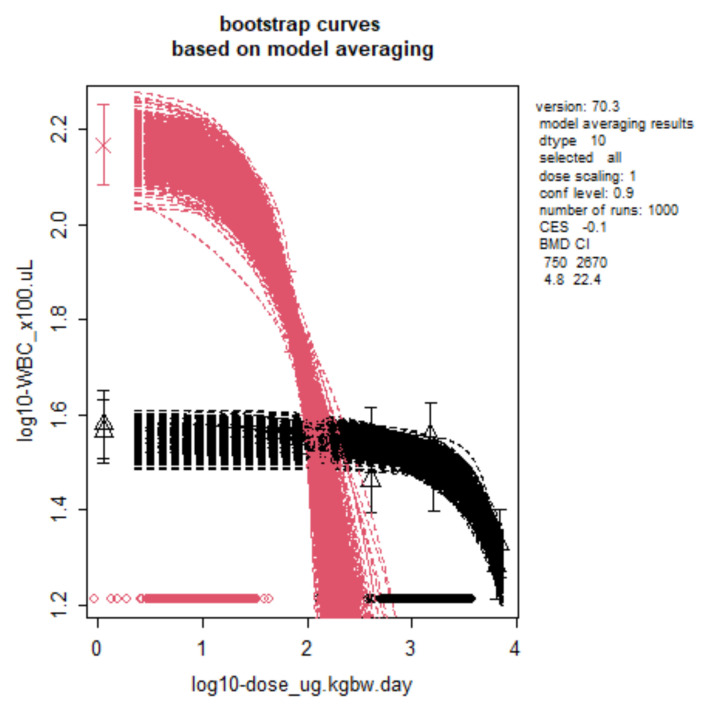
Model averaging results: nivalenol (black line) and T2 toxin (red line).

To omit the difference in the effect sizes that were used to derive the TRV for NIV and T2/HT2 toxin, a simultaneous benchmark dose (BMD) analysis was performed using the PROAST software package version 70.3 with the datasets that were used to derive the BMDLs for NIV and T2/HT2. The BMD analysis of the individual datasets was performed with the current PROAST software and according to the current EFSA BMD guidance [70], which explains the differences with the results from the original BMD analysis by EFSA in 2013 and 2017. The BMD confidence intervals (i.e., BMDL and BMDU) were calculated using all models in the software package and using model averaging (see Appendix E—Figure A2 and Figure A3).

### 4.3. Exposure Assessment

#### 4.3.1. Total Diet Studies

The chronic exposures to the selected single mycotoxins in the assessment groups in this study were previously also calculated for 1–2 year olds, 2–6 year olds and 7–69 year olds by Sprong et al. [27] and Pustjens et al. [26] in the mycotoxin-dedicated TDSs. The sampling, selection and categorization of foods and beverages that were included in the TDSs for 1–2 year olds [26] and 2–6 year olds and 7–69 year olds [27,71] and handling of the concentration data were described in these respective studies.

The mycotoxins considered in those TDSs were analysed using liquid chromatography-tandem mass spectrometry (LC-MS/MS), gas chromatography-tandem mass spectrometry (GC-MS/MS) or immunoaffinity clean-up-high performance liquid chromatography-fluorescence detection (IAC HPLC FLC) [71] (Table A3 and Table A4). In addition, MON was analysed using LC-MS/MS and DAS using GC-MS/MS with the method described in Pustjens et al. (2021). The preparation of the samples differed for the different detection methods [71]. See Sprong et al. [27], Lopez et al. [71] and Pustjens et al. [26] for detailed information regarding the sample preparation and subsequent determination of the mycotoxins. The limits of detection (LOD) and limits of quantification (LOQ) used in the analysis of the lower and upper bound scenarios are given in Appendix C—Table A2 and Table A3.

To obtain the whole distribution of the single and combined exposures, the concentration data obtained from the TDSs were linked to food consumption data from the Dutch national food consumption surveys DNFCS 2007–2010 (7–69 year olds) [72], DNFCS 2005–2006 (2–6 year olds) [73] and the first two years of DNFCS 2012–2016 (1–2 year olds) [74]. In addition, a food conversion table was used to link non-sampled foods to composite samples, proportionally to their composition in the non-sampled foods [27].

The exposure distributions were calculated in a lower bound (LB) and upper bound (UB) scenario. In the LB scenario, the samples that were analysed below the LOD/LOQ were replaced with zero. In the UB scenario, a worst-case scenario, samples analysed below LOD/LOQ were replaced with the value of the LOD or LOQ. Additionally, only for the nephrotoxicity assessment group, where the difference between the LB and UB scenarios was very large, a medium bound (MB) scenario was considered, where the samples analysed below LOD/LOQ were replaced with ½ LOD/LOQ.

#### 4.3.2. Single Compound Exposure Assessment

The exposure to the single mycotoxins was previously also calculated using the Monte Carlo Risk Assessment (MCRA) toolbox (mcra.rivm.nl) by Sprong et al. [27] and Pustjens et al. [26]. MCRA is a modular model and data toolbox developed by RIVM and WUR Biometris to assess the single and combined exposure to chemicals at an individual level [75]. The single mycotoxin exposure estimates were also calculated by us with the observed individual mean (OIM) model in MCRA following the probabilistic approach by Sprong et al. [27] and Pustjens et al. [26]. We confirmed that the results of our calculations were the same as the results from the calculations from the previously published TDSs. We show the results of the single mycotoxin exposure estimates also in Appendix F, Table A5. Since this probabilistic approach generates an uncertainty distribution around each exposure percentile, we used the median values generated as exposure estimates for the respective percentile of the population (this also applies to the results of the combined exposure estimates).

#### 4.3.3. Combined Exposure Assessment

The combined exposure estimates were based on the same data as the single compound exposure assessments (obtained from the TDSs) and also calculated using the OIM model in MCRA. In MCRA, a distribution of the individual co-exposure estimates was derived by expressing the exposure to the selected group of mycotoxins as equivalents of one mycotoxin that was appointed as the reference compound (also called index compound) and subsequently summing those exposures per individual in the food consumption database and dividing these by the individual’s body weight. The exposure was averaged over the number of consumption days per individual. To express the exposure to the mycotoxins as equivalents of the reference compound, the single exposure estimates were scaled to the exposure of the reference compound. The scaling of the compounds was performed using two approaches. (1) It was based on the ratio of the TRVs for the mycotoxins in the selected assessment groups. MCRA calculates these ratios by dividing the TRV of the reference compound by the TRV of the respective mycotoxin in the Risk module of MCRA. These ratios are multiplied with the (individual’s) single exposure estimates to express them as the reference compound. Then, the individual’s combined exposure is calculated by summing the equivalent exposure estimates. Finally, a distribution of all individual combined exposures is produced by MCRA (see Equation (6)).

(6) $${\mathrm{CumulativeExposure}}_{\mathrm{distribution}}=\sum_{i=1}^{I}\mathrm{Ej},i*\;(\mathrm{TRVr}/\mathrm{TRVi})$$

where I is the total number of compounds that are considered in the assessment group, i a considered compound in the assessment group, j is each individual, TRV_r_ the TRV of the selected reference compound, and TRV_i_ the TRV of the considered compound.

(2) Another approach that was taken to combine the exposure to multiple mycotoxins in the final part of the results section was based on RPFs that were calculated for NIV and T2/HT2 toxin (see Section 4.2.1). The RPF was used to express NIV as T2/HT2 toxin equivalents (or vice versa, by taking the inverse of the RPF) before summing the exposure estimates at an individual level and producing a distribution of the individual combined exposures in the Dietary exposure module of MCRA. The following equation shows the implementation of Equation (5) for the example of NIV:

(7) $${\mathrm{Exposure}}_{\mathrm{NIV}\;\mathrm{EQ},j}={\mathrm{exposure}}_{\mathrm{NIV},\;j}+\left({\mathrm{exposure}}_{T2/\mathrm{HT}2,\;j}\cdot {\mathrm{RPF}}_{T2/\mathrm{HT}2}\right)$$

where j is one individual.

### 4.4. Risk Characterisation

The risk metrics for the assessment groups were assessed in two ways. (1) One was by dividing all P50 or all P95 exposure estimates for the single mycotoxin by its identified TRV, which yielded RPQs (Equation (3)). These RPQs were summed at the P50 or at the P95 to obtain a modified reference point index (mRPI) (Equation (4)). This approach is similar to the approach introduced by Vejdovszky et al. [15] and considers RPs specific for a certain organ, rather than the RPs related to the critical effect of the compounds. (2) The second approach was by dividing the P50 and P95 from the distribution of the individual combined exposure estimates by the TRV of the respective reference compound in MCRA (see Section 4.3.3 and Equation (6)). This results in a P50 and P95 individual combined risk quotient for the respective assessment group. Except for the calculations using the RPFs, here the TRVs based on the newly derived BMDLs were manually divided by the combined exposure estimates that were calculated in MCRA using the RPFs. These approaches both assume dose addition of the mycotoxins, and any potential interactions between the mycotoxins are not considered.

## Figures and Tables

**Table 4:** Individual (reference point quotients) and combined risk metrics at common target organ assessment groups– kidney, liver and haematological effects. Median (P50) and high (P95) estimated combined risk for 1–2 year olds, 2–6 year olds and 7–69 year olds in the lower bound (LB) and upper bound (UB) exposure scenarios.

	1–2 Year Olds	1–2 Year Olds	2–6 Year Olds	2–6 Year Olds	7–79 Year Olds	7–79 Year Olds
	P50 (LB-UB))	P95 (LB-UB))	P50 (LB-UB))	P95 (LB-UB)	P50 (LB-UB)	P95 (LB-UB)
Nephrotoxicity						
CIT	(0–0.42)	(0.45–1.0)	(0–0.74)	(0–1.4)	(0–0.34)	(0–1.0)
FB1	(0–0.022)	(0.021–0.088)	(0–0.014)	(0–0.040)	(0–0.023)	(0.005–0.054)
FB2	(0–0.021)	(0–0.084)	(0–0.014)	(0–0.040)	(0–0.023)	(0–0.053)
FB3	(0–0.021)	(0–0.084)	(0–0.014)	(0–0.040)	(0–0.023)	(0–0.053)
NIV	(0.001–0.002)	(0.005–0.005)	(0–0.004)	(0–0.024)	(0–0.002)	(0–0.10)
OTA	(0.023–0.26)	(0.77–1.2)	(0–0.18)	(0.013–0.37)	(0.20–0.30)	(0.70–0.81)
PAT	(0–0.001)	(0–0.025)	(0–0.030)	(0–0.066)	(0–0.012)	(0–0.035)
Common organ risk index mRPI (sum)	(0.024–0.75)	(1.2–2.5)	(0–1.0)	(0.013–2.07)	(0.20–0.72)	(0.71–2.1)
mRPI (individually combined)	(0.06–0.89)	(0.93–2.0)	(0–1.0)	(0.013–1.8)	(0.20–0.75)	(0.70–1.8)
Liver toxicity						
FB1	(0–0.044)	(0.042–0.18)	(0–0.029)	(0–0.080)	(0–0.047)	(0.011–0.11)
FB2	(0–0.041)	(0–0.17)	(0–0.029)	(0–0.080)	(0–0.047)	(0–0.11)
FB3	(0–0.041)	(0–0.17)	(0–0.029)	(0–0.080)	(0–0.047)	(0–0.11)
ZEN	(0.001–0.007)	(0.006–0.018)	(0.001–0.005)	(0.001–0.010)	(0.001–0.003)	(0.001–0.008)
Common organ risk index mRPI (sum)	(0.001–0.13)	(0.048–0.53)	(0–0.092)	(0.001–0.25)	(0.001–0.14)	(0.011–0.33)
mRPI (individually combined)	(0.003–0.14)	(0.044–0.52)	(0.001–0.092)	(0.001–0.25)	(0.001–0.14)	(0.011–0.33)
Haematological effect						
DAS	(0–0.002)	(0–0.004)	(0–0.004)	(0–0.008)	(0–0.003)	(0–0.008)
NIV	(0.005–0.006)	(0.017–0.020)	(0–0.013)	(0–0.092)	(0–0.009)	(0–0.38)
MON	(0–0.21)	(0–0.71)	(0.006–0.085)	(0.018–0.18)	(0.0013–0.034)	(0.007–0.11)
T2 toxin	(0.041–0.20)	(9.5–9.6)	(0.68–2.2)	(7.4–10)	(0.13–0.52)	(1.4–3.3)
HT2 toxin	(0.13–0.87)	(0.47–6.9)	(0–0.21)	(3.2–10)	(0–0.12)	(0.39–1.3)
Common organ risk index mRPI (sum)	(0.18–1.3)	(10–17)	(0.68–2.5)	(11–20)	(0.13–0.68)	(1.8–5.1)
mRPI (individually combined)	(0.29–1.6)	(9.6–16)	(0.69–2.7)	(10–19)	(0.14–0.84)	(1.7–4.5)

**Table 5:** Individual and combined risk metrics at the common effect assessment group of reduced white blood cells. Median (P50) and high (P95) estimated combined risk for 1–2 year olds, 2–6 year olds and 7–69 year olds in the lower bound (LB) and upper bound (UB) exposure scenarios. The combined risk was probabilistically calculated using relative potency factors to combine the exposure and with either nivalenol or T2/HT2 as a reference compound to assess the related risk.

	TRV µg/kg bw/d	1–2 Year Olds	1–2 Year Olds	2–6 Year Olds	2–6 Year Olds	7–79 Year Olds	7–79 Year Olds
		P50 (LB-UB)	P95 (LB-UB)	P50 (LB-UB)	P95 (LB-UB)	P50 (LB-UB)	P95 (LB-UB)
NIV reference compound	1.75	(0.61–2.1)	(15–25)	(1.1–4.1)	(17–30)	(0.21–1.2)	(2.8–7.1)
T2/HT2 reference compound	0.020	(0.38–1.3)	(9.6–16)	(0.68–2.5)	(10–19)	(0.13–0.77)	(1.7–4.5)

**Table 6:** Overview of the model averaged BMD_10_ confidence interval (µg/kg bw/d) of nivalenol and T2/HT2 toxin.

	BMDL_10_	BMDU_10_	BMDU_10_/ BMDL_10_
Nivalenol	750	2700	3.5
T2/HT2 toxin	4.7	22	4.7

BMDL_10_: lower limit of the benchmark dose corresponding to 10% decrease in WBC. BMDU_10_: upper limit of the benchmark dose corresponding to 10% decrease in WBC.

**Table 7:** Individual and combined risk metrics at the common effect assessment group of reduced white blood cells based on the new BMDL_10s_. Median (P50) and high (P95) estimated combined risk for 1–2 year olds, 2–6 year olds and 7–69 year olds in the lower bound (LB) and upper bound (UB) exposure scenarios. The combined risk was probabilistically calculated using relative potency factors to combine the exposure and with either nivalenol or T2/HT2 as a reference compound to assess the related risk.

	TRV New µg/kg bw/d	1–2 Year Olds	1–2 Year Olds	2–6 Year Olds	2–6 Year Olds	7–79 Year Olds	7–79 Year Olds
		P50 (LB-UB)	P95 (LB-UB)	P50 (LB-UB)	P95 (LB-UB)	P50 (LB-UB)	P95 (LB-UB)
NIV as reference compound	3.8	(0.28–0.96)	(7.1–12)	(0.51–1.9)	(7.8–14)	(0.10–0.58)	(1.3–3.3)
T2/HT2 as reference compound	0.024	(0.32–1.1)	(8.1–13)	(0.58–2.2)	(8.9–16)	(0.11–0.66)	(1.5–3.8)

**Table 8:** Overview of mycotoxins in the total diet studies and the mycotoxins included in current assessment.

	Included in Assessment	Not Included in Current Assessment Because	
		Carcinogenic/mutagenic	Not possible to obtain/derive a reference point or RPF for an effect at common target organ or effect
Aflatoxins		x	
Alternaria toxins			x
Beauvericin			x
Citrinin	x		
Deoxynivalenol (group)			x
Diacetoxyscirpenol	x		
Enniatins			x
Ergot alkaloids			x
Fumonisins	x		
Fusarenone-X			x
Moniliformin	x		
Mycophenolic acid			x
Nitropropionic acid			x
Nivalenol	x		
Ochratoxin A	x		
Patulin	x		
Roquefortine C			x
Sterigmatocystin		x	
T2 + HT2 toxin	x		
Zearalenone	x		

## Data Availability

Not applicable.

## Abbreviations

AF	Assessment factor
BMD	Benchmark dose
BMDLx	Lower limit of the benchmark dose related to x% extra risk
BMDUx	Upper limit of the benchmark dose related to x% extra risk
BMR	Benchmark response
CIT	Citrinin
DAS	Diacetoxyscirpenol
DON	Deoxynivalenol
EFSA	European Food Safety Authority
FB	Fumonisin
FusX	Fusarenone-X
HBGV	Health-based guidance value
HI	Hazard index
HQ	Hazard quotient
JECFA	Joint FAO/WHO Expert Committee on Food Additives
LB	Lower bound
LOAEL	Lowest observed adverse effect level
LOD	Limit of detection
LOEL	Lowest observed effect level
LOQ	Limit of quantification
MCRA	Monte Carlo Risk Assessment
MON	Moniliformin
mRPI	Modified Reference Point Index
NAM	New approach methodologies
NIV	Nivalenol
NOAEL	No observed adverse effect level
NPA	Nitropropionic acid
OTA	Ochratoxin A
P50	Median of the distribution
P95	95th percentile of the distribution
PAT	Patulin
RPF	Relative potency factor
PCB	Polychlorinated biphenyls
RPQ	Reference point quotient
RP	Reference point
TDI	Tolerable daily intake
TDS	Total diet study
UB	Upper bound
WBC	White blood cell count
WHO	World Health Organization
ZEN	Zearalenone

## Appendix A

Very few in vivo studies with enniatins and beauvericin were identified. However, fusafungine, a mixture of enniatins, is an anti-inflammatory agent and available as a pharmaceutical [76]. Adverse effects that are reported in the product information sheet include dysgeusia, conjunctival congestion, cough and sneezing, dryness of the nose and throat, and nausea [76]. Since the latest EFSA Scientific Opinion on both compounds, two repeated dose 28-day oral toxicity studies were identified for enniatins [77,78]. In the studies, NOAELs of 0.18 mg/kg bw/d, 1.8 mg/kg bw/d and 20 mg/kg bw/d were derived. These NOAELs were derived specifically for the histomorphometrical effects on thymus, uterus and spleen (females) and enterocyte vacuolization in duodenum (males) and increased reactive oxygen species and reduced glutathione brain levels (males), respectively. However, in the latter study by Okano et al. [77], no adverse effects regarding alterations in haematology, blood biochemistry or histopathology were observed at the end of the study. Maranghi et al. [78] also derived a NOAEL for beauvericin of 1 mg/kg bw/d (females) for increased thyroid pycnotic nuclei and endometrial hyperplasia and of 0.1 mg/kg bw/d (males) for reduced colloid and altered T4 serum levels. In addition, Fraeyman et al. [79] showed that a 21-day exposure to enniatin B did not result in major abnormalities of the liver in broiler chicken, and Manyes et al. [80] also reported no sign of adverse effects in a 28-day repeated dose study in rats. Since no clear indications of nephrotoxicity, liver toxicity or haematoxicity were reported, enniatins and beauvericin were not included in these assessment groups.

Appropriate in vivo studies for other mycotoxins included in the TDSs were not identified in the related EFSA Scientific Opinions or literature after those publication dates. For many mycotoxins, chronic in vivo studies are scarce, and although some mycotoxins were not included in the assessment groups in this study, that does not necessarily mean that they should not be included. New toxicity studies could reveal (new) information and reference points for mycotoxins regarding the specific assessment groups.

### Appendix A.1. Kidney

STC and MON were not included in the kidney assessment group. Some studies indicated that nephrotoxicity might, however, be a relevant outcome for these compounds [49,81,82]. In an acute study with sheep (one dose group) degeneration of the proximal tubules was observed [82], and from a 3-week study in broiler chicken (two dosing moments) degeneration of the tubular epithelial was reported [48]. In acute studies with rats and monkeys, histopathological kidney lesions were observed after administration of STC, in addition to degeneration of the glomeruli and proximal tubules [83]. However, no appropriate, (sub)chronic oral studies with multiple administration doses were identified to derive a reference point based on dose–response data.

### Appendix A.2. Liver

For MON, liver congestions were reported after acute exposure [49]. However, no indications of liver toxicity were reported in the limited identified (sub)chronic studies. Therefore, MON was not included in the liver toxicity assessment group.

Nitropropionic acid (NPA) is a mycotoxin that was included in the TDS for older children and adults, but has not been evaluated by EFSA. Although it was suggested to induce liver toxicity, no in vivo studies were identified that showed liver toxicity after oral administration of NPA.

Although patulin has also been described to induce liver damage, in addition to kidney damage, no (sub)chronic studies of toxicity after oral administration with multiple doses were identified [55].

Danicke [84] reported an overall NOAEL of 3.72 mg ergot alkaloids/kg in the diet of laying hens. While toxic effects included reduced liver weights, proventriculus and gizzard, this study was not used here to derive a TRV for humans. Danicke and Diers [85] also reported reduced liver weights and slightly affected microsomal and mitochondrial liver function in piglets after exposure to ergot alkaloids. This was also not a study that could be used to derive a reference value, as multiple dose moments were lacking. However, the occurrence of liver lesions and other changes in the liver after ergot-contaminated feed intake in chickens and piglets may give rise to additional research into this effect in other animals, including humans [86].

### Appendix A.3. Haematological Endpoints

Although sporadic changes in haematological and clinical chemical endpoints were reported in vivo after DON exposure, no (sub)chronic study was identified that showed a clear adverse effect on haematological parameters, specifically considering a reduction in leukocyte count, after oral exposure to DON [87]. Similarly, another trichothecene type B mycotoxin, fusarenone-X (FusX), was reported to exert stronger emetic toxicity in mink, compared to the other B trichothecenes, DON and NIV [88,89]. This suggests that FusX may also belong to this assessment group; however, no study was identified that reported the in vivo toxicity of FusX on haematological effects.

## Appendix B

**Table A1:** Individual (reference point quotients) and combined risk metrics for common phenomenological effect assessment group– reduced white blood cells. Median and P95 estimated combined risk for 1–2 year olds, 2–6 year olds and 7–69 year olds in the lower bound (LB) and upper bound (UB) exposure scenarios.

	1–2 Year Olds	1–2 Year Olds	2–6 Year Olds	2–6 Year Olds	7–79 Year Olds	7–79 Year Olds
	P50 (LB-UB)	P95 (LB-UB)	P50 (LB-UB)	P95 (LB-UB)	P50 (LB-UB)	P95 (LB-UB)
DAS	(0–0.002)	(0–0.004)	(0–0.005)	(0–0.008)	(0–0.003)	(0–0.008)
NIV	(0.005–0.006)	(0.017–0.020)	(0–0.013)	(0–0.092)	(0–0.009)	(0–0.38)
T2	(0.041–0.20)	(9.5–9.6)	(0.68–2.2)	(7.4—10)	(0.13—0.52)	(1.4—3.3)
HT2	(0.13–0.87)	(0.48–6.9)	(0—0.21)	(3.2—10)	(0—0.12)	(0.39—1.3)
mRPI (sum)	(0.18–0.88)	(10–16)	(0.68–2.4)	(11–20)	(0.13–0.65)	(1.8–5.0)
mRPI (individually combined)	(0.53–1.4)	(9.6–16)	(0.68–2.6)	(10–19)	(0.13–0.80)	(1.7–4.5)

## Appendix C

**Table A2:** Total diet study (TDS) children (2–6 years) and 7–69 years old: Limits of detection (LODs) and quantification (LOQs) of the methods used for the analysis (in µg/kg analysis material, freeze-dried where applicable or in liquid form, for others) of mycotoxins in the relevant food subgroups. Limits of detection (LOD) and limits of quantification (LOQ) of the mycotoxin analyses. From Sprong et al., 2016.

Toxin	^1^ Method	Food Type and Number of Samples (n)	LOD (µg/kg)	LOQ (µg/kg)
PAT	LC-MS/MS	Alcoholic drinks (n = 2) Non-alcoholic beverages (n = 12) Fruit (n = 13) Grains and cereal-based products (n = 9) Nuts and oil seeds (n = 4) Vegetables (n = 14)	6.0 6.0 6.0 6.0 6.0 6.0	20 20 20 20 20 20
DAS HT-2 NIV T-2	GC-MS/MS	Alcoholic beverages (n = 2) Grains and cereal-based products (n = 9) Legumes (n = 2) Soy products (n = 1) Tuber (n = 1)	0.3 0.3 2.0 0.3 0.3	1.0 1.0 5.0 1.0 1.0
CIT FB1 FB2 FB3 MON OTA DAS HT-2 NIV T-2 ZEA	Multimethod LC-MS/MS	Alcoholic drinks (n = 2) Non-alcoholic drinks (n = 12) Confectionary (n = 4) Dairy products (n = 5) Eggs (n = 1) Fish and seafood (n = 2) Fruit (n = 13) Grains and cereal-based products (n = 9) Legumes (n = 2) Meat and offal (n = 6) Nuts and oil seeds (n = 4) Oils and fats (n = 3) Soy products (n = 1) Tuber (n = 1) Vegetables (n = 14)	6.7 3.3 3.3 3.3 6.7 0.2 3.3 6.7 67 1.7 1.7 1.7 1.7 1.7 5.0	20 10 10 10 20 0.5 10 20 200 5.0 5.0 5.0 5.0 5.0 5.0

^1^ IAC-HPLC-FLD: Immunoaffinity column-high pressure liquid chromatography-fluorescence; LC-MS/MS: Liquid chromatography tandem mass spectrometry; GC-MS/MS: Gas chromatography tandem mass spectrometry.

**Table A3:** Total diet study (TDS) toddlers (12–36 months). Limits of detection (LODs) and quantification (LOQs) of the methods used for the analysis (in µg/kg analysis material, freeze-dried where applicable or in liquid form, for others) of mycotoxins in the relevant food subgroups.

Mycotoxin	LOD (LOQ) ^1^ In µg/kg Analysed Material	Method ^2^	Food Type and Number of Samples (n)
PAT	5 (10)	LC-MS/MS	Apple (n = 3) Apple sauce (n = 3) Mushrooms (n = 1) Banana (n = 1) Apple juice (n = 1) Concentrated fruit juices (n = 1)
HT-2 NIV T-2 DAS	0.2 (1) 0.5 (2.5) 0.1 (1) 0.2 (1)	GC-MS/MS	Bread (n = 3) Breakfast cereals (n = 3) Pasta (n = 3) Porridge (n = 3) Rice (n = 3) Legumes (n = 3) Soy products (n = 3) Potatoes (n = 3) Apple juice (n = 1) Concentrated fruit juices (n = 1)
CIT FB1 FB2 FB3 HT-2 MON NIV OTA T-2 ZEA	8 (20) 10 10 10 20 100 50 (200) 0.5 5 3 (5)	Multi-toxin method LC-MS/MS	Bread (n = 3) Breakfast cereals (n = 3) Crackers (n = 3) Pasta (n = 3) Porridge (n = 3) Rice (n = 3) Biscuits (n = 3) Cakes (n = 3) Candies (n = 3) Chocolates (n = 3) Cheeses (n = 3) Creams and ice creams (n = 3) Milk and milk-based beverages (n = 3) Yoghurts and other dairy products (n = 3) Apple (n = 3) Apple sauce (n = 3) Children’s fruits (n = 3) Citrus fruits (n = 3) Dried fruits (n = 3) Other fruits (n = 3) Chicken (n = 3) Mat on bread (n = 3) Offal (n = 3) Pork (n = 3) Sausages (n = 3) Sausages on bread (n = 3) Other juices (n = 3) Soft drinks (n = 3) Syrups (n = 3) Deep-frying fat, (n = 3) Margarines (n = 3) Oils (n = 3) Salty biscuits (n = 3) Brassica vegetables (n = 3) Onion and leek (n = 3) Fruiting vegetables (n = 3) Leafy vegetables, mixed vegetables (n = 3) Other vegetables (n = 3) Root vegetables(n = 3) Tomatoes and tomato products (n = 3) Stem vegetables (n = 2) Beef (n = 1) Mushrooms (n = 1) Banana (n = 1) Apple juice (n = 1) Concentrated fruit juices (n = 1)

^1^ LOD equals the LOQ if no number is mentioned between brackets. ^2^ GC-MS/MS: gas chromatography-tandem mass spectrometry; IAC-HPLC-FLD: immunoaffinity clean-up-high performance liquid chromatography-fluorescence detection; LC-MS/MS: liquid chromatography-tandem mass spectrometry; LOD: limit of detection; LOQ: limit of quantification.

## Appendix D

A four-parameter exponential model was fitted to the (continuous) data (see Table A4, below) plotted against the dose:

(A1) $$y\;=\;a\left(c^{1-e^{\left(-{\left(\frac{x}{b}\right)}^{d}\right)}}\right)$$

In a covariate analysis, parallel curves were fit to the data by applying the same shape parameters (maximum response parameter c and the steepness parameter d) in the four-parameter exponential model to both mycotoxins (T2/HT2 toxin and NIV), but allowing the background (parameter a), the potency (parameter b) and the residual variance to be different between both substances and, in the case of NIV, also between sexes [90,91]. The lowest (most optimal) AIC was reached by using compound and sex as a covariate for parameter a and compound as a covariate on parameter b. No covariate was needed on the residual variance. In other words, the background white blood cell count differed between the NIV and T2/HT2 toxin studies and between males and females in the NIV study. Potency differed between both mycotoxins, but males and females in the NIV study were equally sensitive. The residual variance did not differ between both studies. A good description of the data of each mycotoxin by parallel curves was confirmed by visual inspection (see Figure A1, below).

The RPF is defined as the ratio of two (equipotent) doses, x_NIV_ for the reference compound NIV and x*_T2/HT2_* for T2/HT2 toxin, which both result in the same relative change in WBC response (y):

(A2) $${\mathrm{RPF}}_{T2/\mathrm{HT}2}=\frac{x_{\mathrm{NIV}}}{x_{T2/\mathrm{HT}2}}$$

when assuring the curves of the two mycotoxins are parallel, x/b in the exponential model (Equation (7)) has the same value for both mycotoxins:

(A3) $$\frac{x_{\mathrm{NIV}}}{b_{\mathrm{NIV}}}=\frac{x_{T2/\mathrm{HT}2}}{b_{T2/\mathrm{HT}2}}$$

Combining Equations (A1) and (A2) gives Equation (A3),

(A4) $${\mathrm{RPF}}_{T2/\mathrm{HT}2}=\frac{b_{\mathrm{NIV}}}{b_{T2/\mathrm{HT}2}}$$

which shows that the RPF of T2/HT2 toxin can be directly obtained from its potency parameter b and that of NIV. Calculation of the RPFs and their 90% confidence intervals based on potency parameter b (Figure A1) was performed using PROAST software (see https://www.rivm.nl/en/proast, accessed on 1 October 2021 and https://www.rivm.nl/documenten/proast-manual-menu-version, accessed on 1 October 2021).

**Table A4:** Input data used in the benchmark dose analysis to calculate the relative potency factor of T2/HT2 toxin compared to nivalenol.

Dose µg/kg bw/d	Compound	Sex	WBC ×100/µL	Sd	Sem	n	Comp.Sex
0	NIV	f	38.8	7.7	2.566667	9	niv.f
400	NIV	f	30.5	10.1	3.1939	10	niv.f
1600	NIV	f	29.7	5.5	1.739253	10	niv.f
6400	NIV	f	19.6	4.5	1.423025	10	niv.f
0	NIV	m	38.5	12.2	3.857979	10	niv.m
400	NIV	m	37	12.2	3.857979	10	niv.m
1500	NIV	m	36.5	6.1	1.928989	10	niv.m
6900	NIV	m	21.6	3.9	1.3	9	niv.m
0	T2HT2	m	148.3	NA	7.3	8	T2HT2.m
45	T2HT2	m	89.5	NA	3.6	8	T2HT2.m
68	T2HT2	m	69.2	NA	8.3	8	T2HT2.m
90	T2HT2	m	52	NA	7.3	8	T2HT2.m

f: female, m: male, WBC: white blood cell count, sd: standard deviation, sem: standard error of the mean, n: number of animals in dose group. Note: dose nivalenol is expressed in mg/kg bw/d in the EFSA Scientific Opinion. Note: sem of nivalenol was calculated here from sd and n. Note: WBC of T2 in EFSA Scientific Opinion expressed as x1000/µL.

## Appendix E

The data reported in Table A4 were used to derive model averaged BMD10 confidence intervals according to the EFSA BMD guidance [70] in a covariate analysis.

The results for the four continuous models are plotted in Figure A2. The BMD confidence intervals corresponding to 10% decrease in WBC obtained after model averaging (Figure A3) are reported in Table 6 in the main text. The BMDU/BMDL ratio of less than 10 indicates that even though the BMDL lies well below the lowest administered dose, the BMD can be estimated with sufficient precision.

## Appendix F

**Table A5:** Median (P50) and high (P95) estimated exposure estimates (µg/kg/day) of the single mycotoxins for 1–2 year olds, 2–6 year olds and 7–69 year olds in the lower bound (LB) and upper bound (UB) exposure scenarios.

	1–2 Year Olds	1–2 Year Olds	2–6 Year Olds	2–6 Year Olds	7–79 Year Olds	7–79 Year Olds
	P50 (LB-UB)	P95 (LB-UB)	P50 (LB-UB)	P95 (LB-UB)	P50 (LB-UB)	P95 (LB-UB)
CIT	(0–0.042)	(0.045–0.10)	(0–0.074)	(0–0.14)	(0–0.033)	(0–0.10)
DAS	(0–0.001)	(0–0.003)	(0–0.003)	(0–0.005)	(0–0.002)	(0–0.005)
FB1	(0–0.044)	(0.042–0.18)	(0–0.029)	(0–0.080)	(0–0.047)	(0.011–0.11)
FB2	(0–0.041)	(0–0.17)	(0–0.029)	(0–0.080)	(0–0.045)	(0–0.11)
FB3	(0–0.041)	(0–0.17)	(0–0.029)	(0–0.080)	(0–0.045)	(0–0.11)
HT2 toxin	(0.003–0.009)	(0.011–0.14)	(0–0.004)	(0.063–0.20)	(0–0.002)	(0.008—0.026)
MON	(0–0.21)	(0–0.71)	(0.006–0.085)	(0.018–0.18)	(0.001–0.034)	(0.007–0.11)
NIV	(0.008–0.011)	(0.030–0.036)	(0–0.024)	(0–0.16)	(0–0.016)	(0–0.67)
OTA	(0.001–0.006)	(0.019–0.028)	(0–0.004)	(0.001–0.009)	(0.005–0.007)	(0.017–0.019)
PAT	(0–0.002)	(0–0.098)	(0–0.12)	(0–0.27)	(0–0.047)	(0–0.14)
T2 toxin	(0.001–0.004)	(0.19–0.19)	(0.014–0.043)	(0.15–0.20)	(0.003—0.010)	(0.029—0.067)
ZEN	(0.003–0.024)	(0.020–0.059)	(0.001–0.017)	(0.001–0.033)	(0.001–0.009)	(0.002–0.027)
